# Red cell distribution width to albumin ratio predicts short-term mortality in urosepsis: a dual-cohort study

**DOI:** 10.3389/fnut.2026.1709663

**Published:** 2026-02-10

**Authors:** Ying Yu, Zhenlin He, Wei Li, Kun Wang, Decai Zhu, Lei Zhang, Xiangqian Nie

**Affiliations:** 1 Department of Urology, Zhejiang Provincial People's Hospital Bijie Hospital, Bijie, China; 2 Department of Urology, The Affiliated Hospital of Guizhou Medical University, Guiyang, China

**Keywords:** adverse prognosis, biomarker, red cell distribution width to albumin ratio, risk stratification, urosepsis

## Abstract

**Methods:**

This study retrospectively collected data on patients with urosepsis from the MIMIC-IV database and Bijie Hospital of Zhejiang Provincial People’s Hospital. Multiple statistical methods were employed to explore the association between the RAR and short-term adverse outcomes, including multivariable Cox regression, restricted cubic spline (RCS) regression, and Kaplan–Meier (KM) survival analyses. Subsequently, three machine learning algorithms were utilized to screen for important features, followed by the construction of a multivariable Cox regression model for risk prediction. The performance of the risk prediction model was evaluated using receiver operating characteristic (ROC) curve analysis, with comparative validation performed via DeLong’s test.

**Results:**

This study ultimately included 3,374 patients with urosepsis. The 28-day ICU mortality and in-hospital mortality rates were 15.20 and 13.75%, respectively. In the fully adjusted multivariate models, RAR, whether treated as a continuous or categorical variable, remained significantly associated with both 28-day ICU mortality and in-hospital mortality. For each unit increase in continuous RAR, the hazard ratios (HRs) were 1.10 (95% confidence interval [CI]: 1.05–1.16) and 1.09 (95% CI: 1.04–1.15), respectively. Compared with the low-RAR group, the high-RAR group showed HRs of 1.55 (95% CI: 1.19–2.01) and 1.39 (95% CI: 1.06–1.82) for the two outcomes. RCS analysis indicated a positive dose–response relationship between RAR and short-term adverse prognosis. DeLong’s test and ROC curve analysis demonstrated that RAR can appropriately enhance the predictive ability of routine critical illness scores for adverse outcomes. Moreover, a risk-prediction model incorporating RAR Slightly better than traditional severity scores (such as SOFA and SAPS II) in identifying high-risk patients. All findings were validated in an external cohort.

**Conclusion:**

This study suggests that the RAR could serve as a predictor of short-term mortality risk in patients with urosepsis, with potential for translation into a clinical stratification tool to aid early identification of high-risk patients and guide intervention. However, its clinical utility needs to be further validated in larger prospective studies.

## Background

Sepsis is a complex systemic inflammatory response syndrome triggered by infection, characterized by high mortality rates and recognized as a leading cause of death in non-cardiac intensive care units (ICUs) (1, 2). This syndrome can originate from infections at various sites, each exhibiting distinct pathophysiological features (3–5). Genitourinary tract infections account for 9–31% of sepsis cases, and sepsis resulting specifically from such infections is termed urosepsis. As a severe complication of urinary tract infection (UTI), urosepsis poses a significant threat to individual health and public health systems (6, 7). In recent years, the increasing incidence of urological diseases and related surgical interventions, coupled with escalating antibiotic resistance among pathogenic microorganisms, has further intensified the disease burden associated with urosepsis (6, 7). In this context, in-depth investigation of risk factors for UTI-related bloodstream infections and identifying reliable early biomarkers for high-risk populations are critical for enhancing preventive measures and optimizing therapeutic strategies.

Unfortunately, although some scoring systems have been shown to correlate with outcomes in patients with urosepsis (8–10), their reliance on numerous parameters renders them cumbersome for routine use, limiting their utility as satisfactory predictive tools in clinical practice. Consequently, there is a pressing need for convenient biomarkers with strong predictive power to assist clinicians in identifying high-risk patients and guiding treatment decisions. Previous studies have demonstrated that a patient’s nutritional status, along with levels of inflammation and oxidative stress, significantly influences both the risk of developing urosepsis and its clinical outcomes (11, 12), underscoring the importance of monitoring these parameters. Serum albumin serves as a key biomarker for assessing nutritional and inflammatory status, fulfilling multiple roles including anti-inflammatory and antioxidant functions, as well as osmotic regulation (13, 14). Simultaneously, the red cell distribution width (RDW), a routine parameter reflecting heterogeneity in erythrocyte volume, is elevated in diverse pathological states such as inflammation, oxidative stress, and malnutrition, and is regarded as a composite indicator of multidimensional physiological dysfunction (15, 16). Together, these two markers reflect the intertwined pathophysiological mechanisms involving inflammation, nutrition, and oxidative stress.

Collectively, these markers reflect the intertwined pathophysiological mechanisms linking inflammation, nutrition, and oxidative stress. Leveraging their complementary roles, researchers have proposed the red cell distribution width-to-albumin ratio (RAR) as a composite index (17). Unlike traditional scoring systems such as SOFA or SAPS-II, RAR may indirectly reflect a patient’s inflammatory, nutritional, and oxidative stress status, offering an integrated model of the pathophysiology in critically ill patients (18). Utilizing routine laboratory measurements (RDW and albumin), RAR eliminates the need for specialized testing. Furthermore, existing evidence suggests that RAR holds predictive value for adverse clinical outcomes in patients with various infection-related critical illnesses (18–22); however, its potential significance specifically in urosepsis remains unclear. Given the distinct pathophysiological features of sepsis originating from different sources (3–5), further investigation into the role of RAR in prognostic assessment for urosepsis patients is of considerable importance. Therefore, this study aims to explore the association between RAR and adverse clinical outcomes in patients with urosepsis.

## Methods

### Data sources

This study utilized data from two retrospective cohorts. The internal cohort comprised patients sourced from the publicly available Medical Information Mart for Intensive Care IV (MIMIC-IV), maintained by the Massachusetts Institute of Technology (23). Under the database access agreement, researchers are authorized to use its de-identified clinical data. This retrospective analysis of anonymized data was granted an exemption from ethics review. The external real-world cohort consisted of critically ill patients with urosepsis admitted to the Departments of Urology and Critical Care Medicine at Zhejiang Provincial People’s Hospital between January 2020 and January 2025. The construction and use of this cohort were approved by the hospital’s Institutional Ethics Committee.

### Study population

This study focused on patients admitted to the ICU with sepsis originating from a UTI. Patient diagnosis was based on criteria established in previous literature (24, 25). Sepsis was defined according to the Sepsis-3 consensus criteria, requiring an increase in the Sequential Organ Failure Assessment (SOFA) score by ≥2 points from baseline. The diagnostic criteria for UTI differed between the two cohorts: the internal cohort was identified using International Classification of Diseases (ICD) diagnostic codes from the MIMIC-IV database, while the external cohort was confirmed by typical clinical presentation (pyuria and/or bacteriuria on urinalysis) and a positive urine culture. Only patients with an initial admission diagnosis of UTI who also met the Sepsis-3 criteria were enrolled in the UTI-sepsis cohort. All enrolled patients met the following criteria: (1) age ≥18 years, (2) ICU stay of at least 24 h, and (3) availability of complete serum lactate and albumin measurements at ICU admission.

### Variable extraction and endpoint definition

Following the acquisition of necessary permissions, data extraction was performed using PostgreSQL (version 13.7.2) and Navicat Premium (version 16.0) tools in conjunction with Structured Query Language (SQL). The extracted variables were categorized into six groups: (1) demographic data; (2) comorbidities; (3) vital signs; (4) laboratory test indicators; (5) disease severity scores; and (6) administered treatments. A detailed description of each variable is provided in Supplementary Tables S1–S3. The RAR was calculated according to established methodology (17, 20–22), using the formula: RDW (%)/serum albumin (g/dL).

To handle missing data, variables with more than 30% missing values were excluded from the analysis. For the remaining variables included in the analysis that still contained missing data, multiple imputation was performed using the “mice” package (version 3.16.0) in R. The imputation model incorporated all variables used in the primary analysis: continuous variables requiring imputation, complete primary exposure variables (RAR and its components), complete outcome variables (28-day mortality), and all other complete categorical and continuous covariates. This approach enhances the plausibility of the Missing at Random (MAR) assumption by leveraging all available information to predict missing values.

### Association between RAR and endpoints

Cox proportional hazards regression models were employed to evaluate the association between the red cell distribution width-to-albumin ratio (RAR) and clinical endpoints. To control for potential confounders, three sequentially adjusted models were constructed: Model 1 was unadjusted; Model 2 was adjusted for demographic characteristics (age, gender, race, and weight) and baseline comorbidities that differed between groups (survivors vs. non-survivors); Model 3 was further adjusted for variables that showed significant differences between survivors and non-survivors (for details, see Supplementary Tables S1–S3). To mitigate the impact of multicollinearity on model stability, the variance inflation factor (VIF) was calculated for all variables in Model 3, and variables with VIF > 5 were excluded. Subsequently, restricted cubic splines (RCS) with four knots (at the 5th, 35th, 65th, and 95th percentiles) were used to explore possible nonlinear relationships between RAR and the endpoints. Kaplan–Meier (KM) survival curves were generated for supplementary visualization.

### Risk prediction modeling and validation

The internal cohort was first randomly split into a training set and a validation set at a ratio of 7:3. In the training set, three machine learning algorithms, Random Forest, Gradient Boosting, and Boruta feature ranking, were used to identify key variables significantly associated with patient prognosis. Subsequently, multivariate Cox regression was applied to select independent predictors for constructing a risk prediction model. The risk score was calculated using the following formula: Risk Score = (Variable₁ × *β*₁) + (Variable₂ × *β*2) + ⋯ + (Variablen × βn), where β denotes the regression coefficient. The predictive performance of the model was evaluated using receiver operating characteristic (ROC) curves and the area under the curve (AUC). Furthermore, its stability was assessed in an external validation cohort with differing patient characteristics.

## Statistical analysis

Continuous variables are expressed as mean ± standard deviation (SD), and differences between groups were analyzed using Student’s *t*-test or analysis of variance (ANOVA), as appropriate. Categorical variables are presented as numbers (percentages), and group differences were analyzed using Pearson’s chi-square test or Fisher’s exact test. All statistical analyses were performed using R software (version 4.5.1). A two-sided *p*-value < 0.05 was considered statistically significant. For data visualization and comparative analysis, patients were divided into two groups (low risk and high risk), based on the median RAR value. It is important to emphasize that this classification was used solely for descriptive and exploratory purposes and does not imply the existence of clinically validated risk thresholds. DeLong’s test was employed to determine whether RAR provided statistically significant incremental value over six traditional severity scores.

## Results

### Baseline patient data

A total of 3,374 critically ill patients with sepsis were included in the analysis based on stringent inclusion criteria. The mean age of the cohort was 70.4 years, and 1,480 (43.9%) were male. The baseline characteristics stratified by RAR are presented in Table 1. Compared to patients with lower RAR, those with higher RAR were generally older, had more comorbidities (e.g., acute kidney injury, chronic kidney disease, dyslipidemia, chronic obstructive pulmonary disease), and more frequently received vasopressors, corticosteroids, and continuous renal replacement therapy.

**Table 1:** Baseline data of patients in RAR grouping.

Variable	ALL	Low	High	*p* value
	*N* = 3,374	*N* = 1,687	*N* = 1,687	
RAR	5.75 (1.72)	4.47 (0.59)	7.03 (1.52)	0.000
Age	70.4 (15.1)	70.9 (15.2)	69.9 (14.9)	0.050
Gender	1,480 (43.9%)	755 (44.7%)	725 (43.0%)	0.329
Race	2,196 (65.1%)	1,099 (65.1%)	1,097 (65.1%)	1.000
Weight	82.1 (25.6)	81.9 (24.7)	82.2 (26.4)	0.738
HPY	1,224 (36.3%)	688 (40.8%)	536 (31.8%)	<0.001
AKI	2,073 (61.4%)	906 (53.7%)	1,167 (69.2%)	<0.001
CKD	951 (28.2%)	447 (26.5%)	504 (29.9%)	0.030
DM	1,263 (37.4%)	615 (36.4%)	648 (38.4%)	0.244
HLD	1,181 (35.0%)	664 (39.3%)	517 (30.7%)	<0.001
HF	1,304 (38.6%)	648 (38.4%)	656 (38.9%)	0.783
MI	351 (10.4%)	191 (11.3%)	160 (9.49%)	0.093
IHD	1,235 (36.6%)	630 (37.3%)	605 (35.9%)	0.406
COPD	547 (16.2%)	247 (14.6%)	300 (17.8%)	0.015
SOFA	6.51 (3.59)	5.79 (3.36)	7.23 (3.67)	<0.001
APSII	57.0 (21.2)	51.4 (19.6)	62.6 (21.4)	<0.001
SAPSII	43.9 (13.5)	41.5 (12.8)	46.4 (13.7)	<0.001
OASIS	35.3 (8.37)	34.5 (8.27)	36.2 (8.39)	<0.001
Charlson	6.27 (2.90)	6.02 (2.86)	6.53 (2.92)	<0.001
APACHEII	21.2 (7.11)	19.9 (6.92)	22.5 (7.05)	<0.001
HR	91.1 (21.2)	88.5 (20.2)	93.7 (21.7)	<0.001
NBPS	121 (25.7)	125 (26.1)	117 (24.7)	<0.001
NBPD	67.9 (19.7)	69.2 (19.1)	66.6 (20.2)	<0.001
RR	20.1 (6.32)	19.7 (6.02)	20.5 (6.58)	<0.001
Spo2	96.6 (4.53)	96.5 (4.63)	96.7 (4.43)	0.319
Hb	10.2 (2.21)	10.9 (2.16)	9.48 (2.03)	<0.001
PLT	204 (118)	204 (102)	203 (132)	0.800
RDW	16.0 (2.57)	14.7 (1.63)	17.3 (2.63)	<0.001
RBC	3.42 (0.78)	3.63 (0.75)	3.22 (0.76)	<0.001
WBC	13.6 (14.2)	12.6 (10.3)	14.6 (17.1)	<0.001
ALB	2.93 (0.61)	3.32 (0.46)	2.53 (0.47)	0.000
AG	15.6 (4.73)	15.6 (4.58)	15.5 (4.87)	0.476
Ca	8.29 (0.96)	8.52 (0.85)	8.07 (1.01)	<0.001
Cl	104 (7.94)	103 (7.38)	104 (8.43)	<0.001
Glu	154 (84.3)	158 (84.0)	149 (84.4)	0.001
K	4.22 (0.80)	4.21 (0.78)	4.23 (0.82)	0.457
Sodium	138 (6.72)	138 (6.26)	139 (7.15)	0.488
TCO2	24.1 (6.39)	24.6 (6.12)	23.6 (6.61)	<0.001
Lac	2.36 (1.96)	2.21 (1.80)	2.52 (2.11)	<0.001
PCO2	41.9 (12.3)	42.2 (12.4)	41.5 (12.2)	0.120
PH	7.35 (0.10)	7.36 (0.10)	7.35 (0.10)	<0.001
PO2	119 (97.2)	125 (100)	113 (93.8)	0.001
INR	1.67 (1.04)	1.53 (0.86)	1.81 (1.18)	<0.001
PT	18.1 (10.6)	16.6 (8.80)	19.5 (12.0)	<0.001
PTT	39.9 (24.4)	38.5 (24.7)	41.3 (24.1)	0.001
ALT	130 (609)	154 (744)	107 (432)	0.025
AST	218 (1,030)	243 (1,190)	193 (841)	0.159
TB	2.07 (5.01)	1.37 (3.42)	2.77 (6.12)	<0.001
CRE	1.83 (1.71)	1.68 (1.60)	1.99 (1.81)	<0.001
URE	37.0 (29.0)	32.5 (26.1)	41.4 (30.9)	<0.001
SA	2,310 (68.5%)	1,143 (67.7%)	1,167 (69.2%)	0.367
VP	2,142 (63.5%)	1,004 (59.5%)	1,138 (67.5%)	<0.001
GC	1,090 (32.3%)	482 (28.6%)	608 (36.1%)	<0.001
Ventilation	2,893 (85.7%)	1,477 (87.5%)	1,416 (84.0%)	0.004
CRRT	326 (9.66%)	124 (7.35%)	202 (12.0%)	<0.001
Hosp dead	464 (13.8%)	171 (10.1%)	293 (17.4%)	<0.001
Hosp 28 time	19.6 (19.6)	19.0 (19.0)	20.2 (20.3)	0.091
ICU dead	513 (15.2%)	188 (11.1%)	325 (19.3%)	<0.001
ICU 28 time	7.15 (8.69)	7.45 (8.69)	6.86 (8.69)	0.049

Patients in the high-RAR group exhibited less stable vital signs, characterized by faster heart rates, higher respiratory rates, and lower systolic and diastolic blood pressures. Furthermore, significant differences were observed in numerous laboratory parameters between the two groups. Higher RAR was associated with elevated levels of RDW, WBC, chloride, lactate, INR, PT, PTT, total bilirubin, creatinine, and blood urea nitrogen. Conversely, patients in the high-RAR group had lower levels of hemoglobin, red blood cell count, albumin, serum calcium, serum glucose, total carbon dioxide combining power, pH, partial pressure of oxygen, and ALT.

All clinical severity scores (SOFA, APSII, SAPSII, OASIS, Charlson, APACHEII) were significantly higher in the high-RAR group, indicating greater illness severity. Ultimately, both the 28-day ICU mortality (19.3% vs. 11.1%, *p* < 0.001) and the 28-day in-hospital mortality (17.4% vs. 10.1%, *p* < 0.001) were significantly higher in the high-RAR group compared to the low-RAR group.

### Association between RAR and short-term mortality in critically ill patients with Urosepsis

As shown in Tables 2, a higher RAR was significantly associated with increased 28-day ICU mortality across all three Cox regression models: the unadjusted model (Model 1: HR = 1.22, 95% CI 1.17–1.26, *p* < 0.001), the model adjusted for demographics and comorbidities (Model 2: HR = 1.20, 95% CI 1.15–1.25, *p* < 0.001), and the fully adjusted model (Model 3: HR = 1.10, 95% CI 1.05–1.16, *p* < 0.001). Similarly, the high-RAR group exhibited a significantly elevated mortality risk compared to the low-RAR group in all models (Model 1: HR = 1.90, 95% CI 1.59–2.28, *p* < 0.001; Model 2: HR = 1.68, 95% CI 1.40–2.02, *p* < 0.001; Model 3: HR = 1.25, 95% CI 1.01–1.49, *p* = 0.039).

**Table 2:** The relationship between RAR and short-term mortality rate in ICU (discovery queue).

Characteristic	Model 1			Model 2			Model 3		
	HR	95% CI	*p*-value	HR	95% CI	*p*-value	HR	95% CI	*p*-value
RAR	1.22	1.17, 1.26	<0.001	1.20	1.15, 1.25	<0.001	1.10	1.05, 1.16	<0.001
RAR group									
Low	Ref	Ref		Ref	Ref		Ref	Ref	
High	1.90	1.59, 2.28	<0.001	1.68	1.40, 2.02	<0.001	1.25	1.01, 1.49	0.039

CI, Confidence Interval; HR, Hazard Ratio.

**Figure 1:**
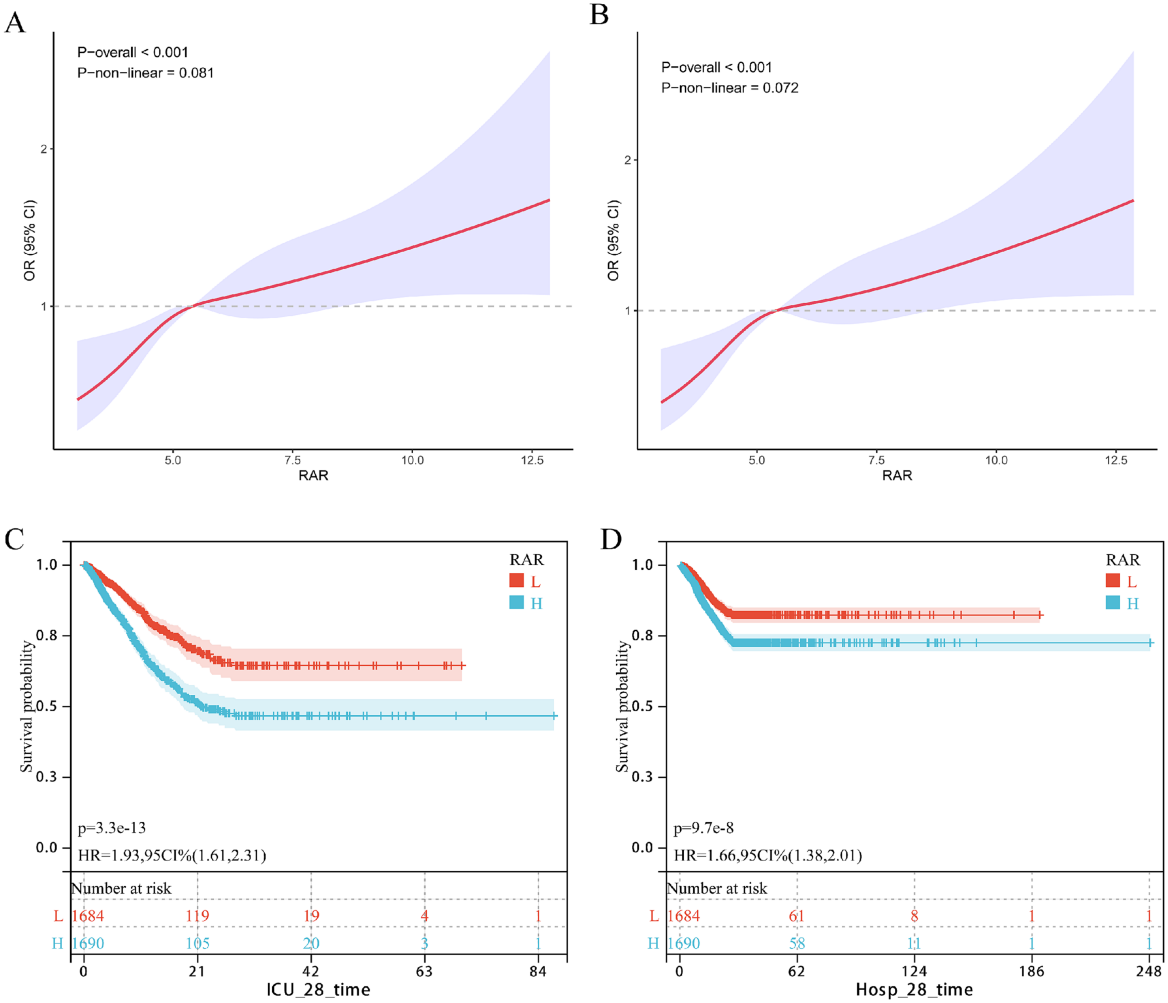
The correlation between RAR and short-term mortality in patients with urinary sepsis (internal discovery queue). **(A, B)** Dose response curves of RAR and short-term ICU/Hosp mortality, **(A)** ICU, **(B)** Hosp; **(C, D)** KM survival curve of RAR and ICU/Hosp short-term mortality, **(C)** ICU, **(D)** Hosp.

Consistent findings were observed for in-hospital mortality (Table 3). A higher RAR remained associated with greater risk (Model 1: HR = 1.18, 95% CI 1.14–1.23, *p* < 0.001; Model 2: HR = 1.17, 95% CI 1.12–1.22, *p* < 0.001; Model 3: HR = 1.09, 95% CI 1.04–1.15, *p* = 0.001), and the high-RAR group consistently had higher mortality than the low-RAR group (Model 1: HR = 1.65, 95% CI 1.37–2.00, *p* < 0.001; Model 2: HR = 1.49, 95% CI 1.23–1.81, *p* < 0.001; Model 3: HR = 1.21, 95% CI 1.04–1.38, *p* = 0.027). Furthermore, restricted cubic spline (RCS) analysis revealed a positive dose–response relationship between RAR and short-term mortality in patients with severe urosepsis (Figures 1A,B). Kaplan–Meier survival curves confirmed that a higher RAR was associated with significantly lower short-term survival probability (Figures 1C,D).

**Table 3:** The relationship between RAR and short-term mortality rate in hospital (discovery queue).

Characteristic	Model 1			Model 2			Model 3		
	HR	95% CI	*p*-value	HR	95% CI	*p*-value	HR	95% CI	*p*-value
RAR	1.18	1.14, 1.23	<0.001	1.17	1.12, 1.22	<0.001	1.09	1.04, 1.15	0.001
RAR group									
Low	Ref	Ref		Ref	Ref		Ref	Ref	
High	1.65	1.37, 2.00	<0.001	1.49	1.23, 1.81	<0.001	1.21	1.04, 1.38	0.027

CI, Confidence Interval; HR, Hazard Ratio.

### Incremental effect of RAR

**Figure 2:**
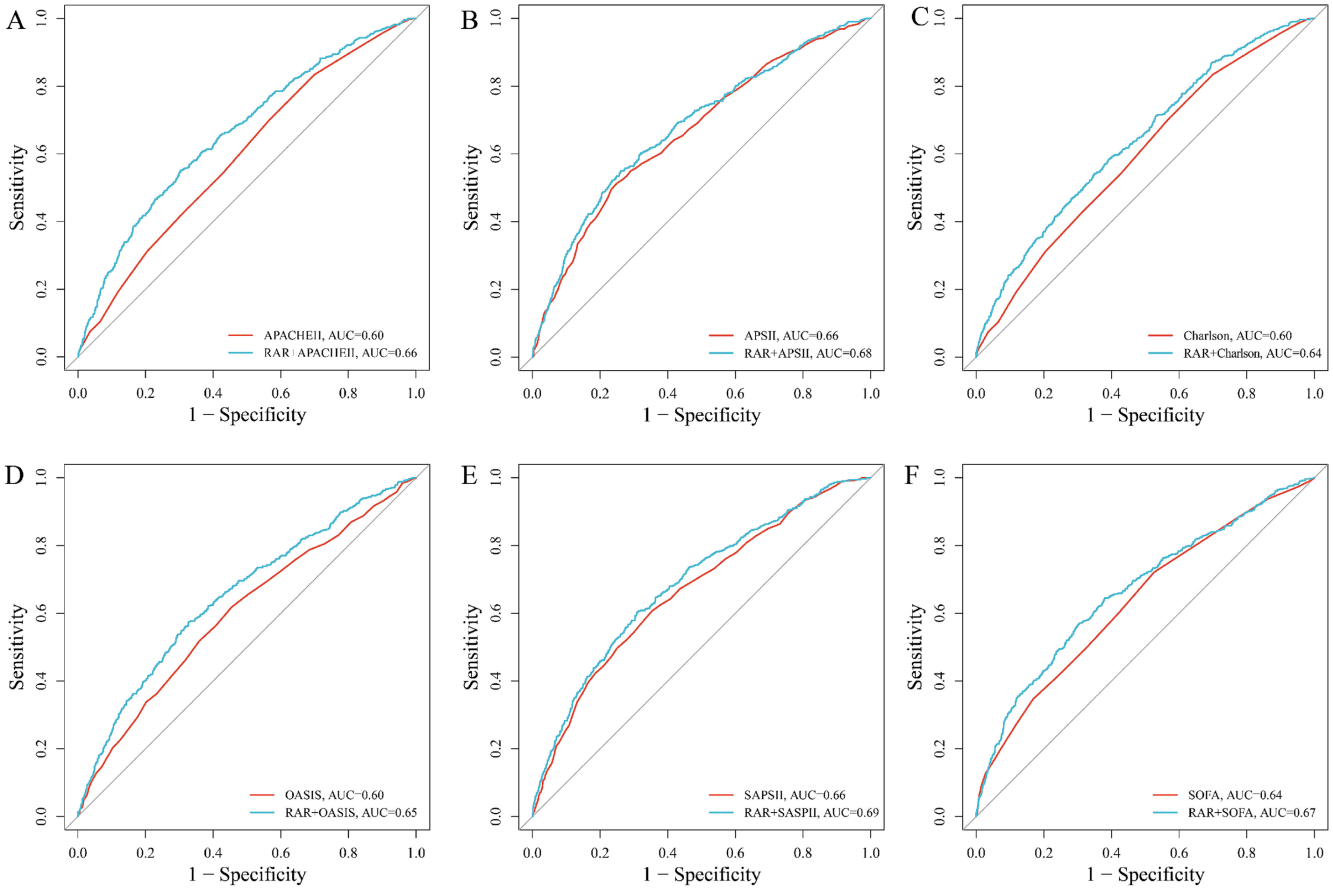
The incremental effect of RAR **(A–F)**: ROC curve, **(A)** SOFA, **(B)** APSII, **(C)** SAPSII, **(D)** OASIS, **(E)** Charlson, **(F)** APACHE II.

We used AUC analysis to evaluate the impact of adding RAR to existing disease severity scores (SOFA, APS III, SAPS II, OASIS, Charlson, APACHE II) for predicting 28-day ICU mortality. As shown in Figures 2A–F, the inclusion of RAR consistently improved the predictive performance of all scores: APACHE II (0.60–0.66, *P* for DeLong test < 0.001; Figure 2A), APS II (0.66–0.68, *P* for DeLong test = 0.039; Figure 2B), Charlson (0.60–0.63, *P* for DeLong test < 0.001; Figure 2C), OASIS (0.60–0.65, *P* for DeLong test < 0.001; Figure 2D), SAPS II (0.66–0.69, *P* for DeLong test = 0.003; Figure 2E), and SOFA (AUC increased from 0.64 to 0.67, *P* for DeLong test < 0.001; Figure 2F).

### Subgroup and interaction analysis

To investigate whether demographic characteristics and comorbidities influence the association between RAR and 28-day ICU/in-hospital mortality, subgroup analyses and interaction tests were performed. After multivariate adjustment, the results (Supplementary Figures S1, S2) indicated that the association between RAR and short-term mortality in urosepsis patients remained consistent across all demographic and comorbidity subgroups. Interaction tests further showed no significant interaction effects between RAR and any of the aforementioned factors (all *P* for interaction > 0.05).

### External cohort validation

**Figure 3:**
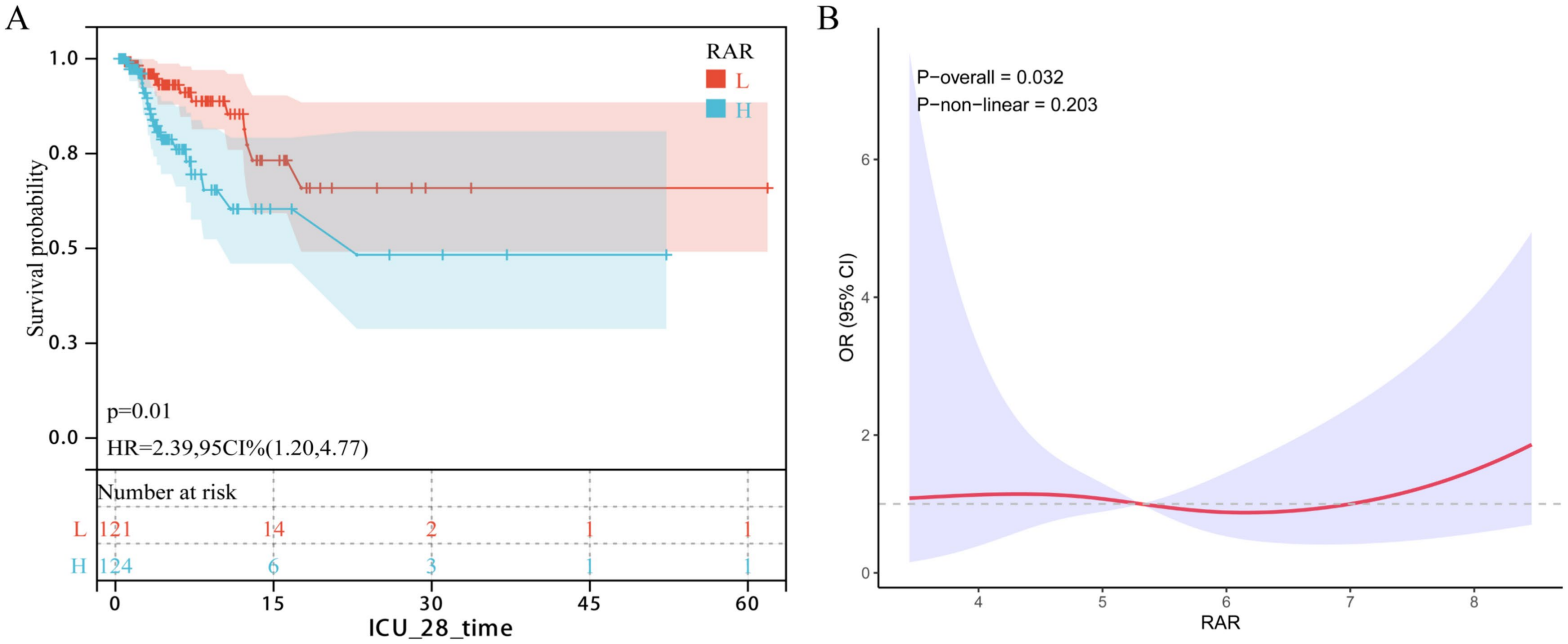
The correlation between RAR and short-term mortality in patients with urinary sepsis (external reality queue). **(A)** KM survival curve, **(B)** RCS curve.

The external validation cohort included 245 patients with urosepsis (28-day mortality = 14.28%). In the Cox regression model adjusted for all potential covariates, a higher RAR score was significantly associated with an increased risk of 28-day mortality (HR = 1.29; 95% CI: 1.00–1.66, *p* = 0.049). Subsequent Kaplan–Meier analysis indicated that patients with RAR scores above the cohort median had significantly higher short-term mortality (log-rank *p* = 0.01; HR = 2.39, 95% CI: 1.20–4.77; Figure 3A). Furthermore, the RCS curve confirmed a significant positive dose–response relationship between RAR and ICU short-term mortality (*p* = 0.032; Figure 3B).

### Development of an RAR-associated risk prediction model

**Figure 4:**
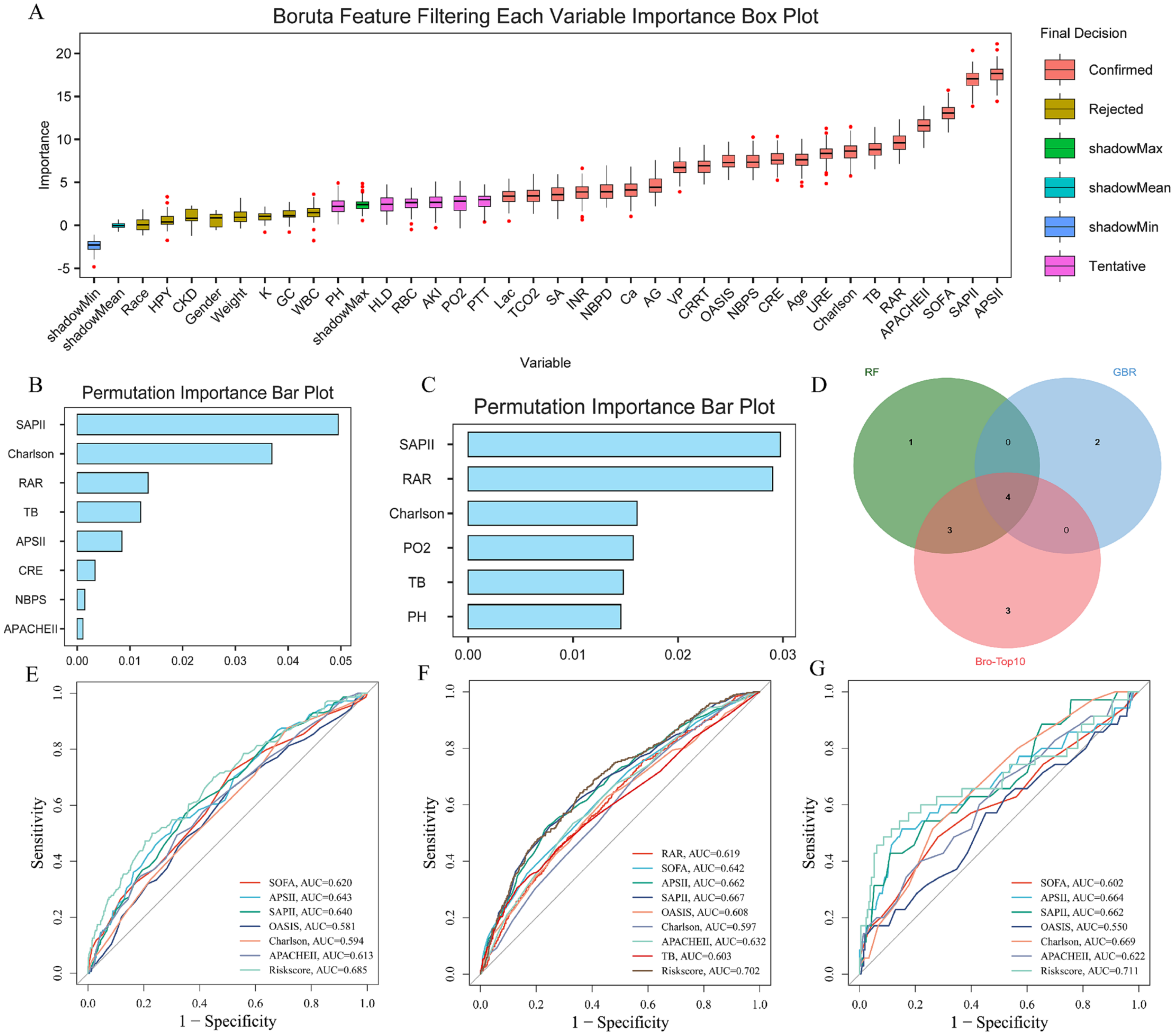
Establishment and evaluation of RAR related prediction analysis model. **(A–D)** The establishment process of RAR related predictive analysis model: **(A)** Boruta feature importance ranking, **(B)** features obtained by random forest algorithm, **(C)** features obtained by gradient elevator, **(D)** acquisition of common important features; **(E–G)** evaluation of risk models in three queues: **(E)** Internal training queue, **(F)** internal verification queue, **(G)** external reality queue.

In the internal training cohort, we employed Random Forest, Gradient Boosting Machine, and the Boruta feature ranking algorithm to identify four important variables: TB, RAR, Charlson Comorbidity Index, and SAPS II score (Figures 4A–D). Subsequent multivariate Cox regression analysis confirms TB, RAR, and SAPS II as independent predictors. A risk assessment model for predicting 28 day ICU mortality in crisis. Strictly ill patients with urosepsis were constructed as follows: Risk Score=(0.03136447 × TB)+(0.26474404 × RAR)+(0.01406001 × RAR) SAPS II) Compared to traditional severity scoring systems (e.g., SOFA, APS III, SAPS II, OASIS, Charlson, APACHE II), this model demonstrated superior sensitivity and specificity in predicting 28-day ICU mortality. The AUC values were 0.685 in the internal training cohort (Figure 4E), 0.702 in the internal validation cohort (Figure 4F), and 0.711 in the external real-world cohort (Figure 4G). These consistent results indicate that the model possesses robust stability and predictive performance across diverse cohorts.

## Discussion

Urosepsis is a severe complication of urinary tract infection, and its incidence has continued to rise in recent years. This trend is closely linked to the increasing prevalence of urinary system diseases and surgeries, as well as growing microbial antibiotic resistance, posing significant challenges to both patients and public health systems (1, 2, 6, 7). Previous studies have indicated that identifying reliable and clinically applicable biomarkers is of great value for improving outcomes in these patients (8–12). This study is the first to establish a significant association between elevated RAR and adverse prognosis in patients with urosepsis. The results demonstrate that a high RAR is an independent risk factor for both 28-day ICU mortality and in-hospital all-cause mortality. Furthermore, we identified a linear positive dose–response relationship between RAR and early mortality, and this association remained consistent across various demographic characteristics and comorbidity statuses, indicating robust findings. Based on these results, we developed a simple yet effective risk stratification tool using routinely available clinical parameters, which may assist healthcare providers in early identification of high-risk patients and timely intervention.

The RDW is a routine hematological parameter that reflects heterogeneity in red blood cell volume. In recent years, RDW has been widely used to assess inflammation and nutritional status in critically ill patients (15, 16). The underlying mechanism involves the hyperinflammatory state in conditions such as urosepsis, which suppresses erythropoietin production and disrupts erythrocyte maturation, leading to an increased proportion of immature red cells in circulation (26, 27). Inflammatory cytokines also impair iron utilization, promote erythrocyte apoptosis, and may alter cell membrane stability and ion channels, thereby changing red cell morphology (28, 29). Collectively, these pathological changes increase heterogeneity in red cell volume, reflected as an elevated RDW.

On the other hand, albumin is the major plasma protein synthesized by the liver and plays key roles in maintaining colloid osmotic pressure, antioxidation, and anti-inflammatory processes (13, 14). Serum albumin levels are also a common indicator of nutritional status and systemic inflammation. Mechanistically, hypoalbuminemia and inflammation exhibit a bidirectional relationship: hypoalbuminemia (often reflecting malnutrition) is associated with a systemic hyperinflammatory state, while persistent inflammation further suppresses albumin synthesis, exacerbating malnutrition and creating a vicious cycle (30–33). Moreover, hypoalbuminemia can promote oxidative stress, lipid peroxidation, immune dysregulation, and programmed cell death, collectively worsening tissue and organ injury in sepsis (34–36). Given the complexity of critical illness, a single biomarker is often insufficient for accurate prognosis. Since RDW and albumin reflect common pathophysiological processes, such as inflammation, nutritional status, and oxidative stress, that are central drivers of adverse outcomes in critically ill patients, the RAR has recently been proposed as a composite biomarker. This integrated measure aims to provide a more comprehensive assessment of clinical risk and prognosis.

As a composite index derived from routine laboratory tests, the RAR has been shown to provide integrated clinical information beyond single parameters and has been applied for prognostic assessment in various infection-related critical illnesses, including community-acquired bacteremia and sepsis (18–22). However, sepsis from different infectious sources exhibits distinct pathophysiological characteristics: pulmonary infections are marked primarily by alveolar-capillary barrier disruption and macrophage overactivation, frequently progressing to acute respiratory distress syndrome; abdominal infections are characterized by bacterial translocation and mixed pathogen-associated molecular patterns, often accompanied by intra-abdominal hypertension; whereas urinary tract infections typically trigger a systemic inflammatory response via Gram-negative endotoxins and early kidney injury (3–5). These differences underscore the importance of individualized, infection source-based precision medicine in sepsis management. Currently, the potential value of RAR in the clinical management of urosepsis patients remains unclear.

Against this background, our study is the first to demonstrate that an elevated RAR is an independent risk factor for both 28-day ICU mortality and in-hospital all-cause mortality in patients with urosepsis. This association remained consistent across various demographic characteristics and comorbidities, indicating that RAR may serve as an effective predictive marker for adverse outcomes in this population. Furthermore, we developed and validated a risk prediction model incorporating routine clinical indicators and the RAR to estimate the 28-day ICU mortality risk in urosepsis patients. Compared to traditional severity scores, our model demonstrated higher sensitivity and specificity in both internal and external validation cohorts, with stable and reliable predictive performance. These findings suggest that this model could facilitate individualized risk stratification and guide critical clinical decision-making, thereby providing a basis for improving outcomes in urosepsis patients.

While this study provides clinically meaningful findings and develops a potentially useful risk prediction model, several limitations should be acknowledged. First, despite employing rigorous statistical methods—including multivariable Cox regression, Kaplan–Meier survival analysis, and dose–response curves, and validating the results in an independent real-world cohort, all data of independent real-world cohort were derived from a single tertiary hospital with a limited sample size, which may affect the generalizability of the findings. Second, although we controlled for numerous confounders in our analysis, residual confounding from variables such as the type of urological surgery, specific pathogens, and factors influencing albumin levels (e.g., albumin infusion or antibiotic use) could still affect the observed association between RAR and mortality. Third, evolving definitions of sepsis based on updated clinical guidelines may have introduced variability in case inclusion and comparability across different time periods. Similarly, the inability to definitively ascertain the infection source in some patients may have introduced heterogeneity into the study population. Finally, given the retrospective observational design, our study cannot establish a causal relationship between RAR and adverse outcomes in urosepsis patients. Based on these limitations, future large-scale, multicenter, prospective studies with more comprehensive clinical data are warranted to further validate the prognostic value and clinical utility of RAR in this population.

## Conclusion

In conclusion, this study demonstrates that an elevated RAR is associated with short-term mortality in critically ill patients with urosepsis. Incorporating the RAR into traditional severity scores could improve the accuracy of prognostic assessment and aid in the early identification of high-risk patients. Furthermore, the observed linear relationship between RAR and mortality suggests its utility in refining risk stratification. As an easily accessible metric, RAR shows promise as a supplementary biomarker; however, its clinical utility requires further validation in prospective, multicenter studies.

## Data Availability

The datasets presented in this study can be found in online repositories. The names of the repository/repositories and accession number(s) can be found in the article/Supplementary material.
