# Supplementary material for: Red cell distribution width to albumin ratio predicts short-term mortality in urosepsis: a dual-cohort study
Source: Front Nutr. 2026 Feb 10;13:1709663. doi: 10.3389/fnut.2026.1709663 (PMC12929096; doi:10.3389/fnut.2026.1709663)
Supplement: Supplementary file 1 [file Supplementary_file_1.docx]

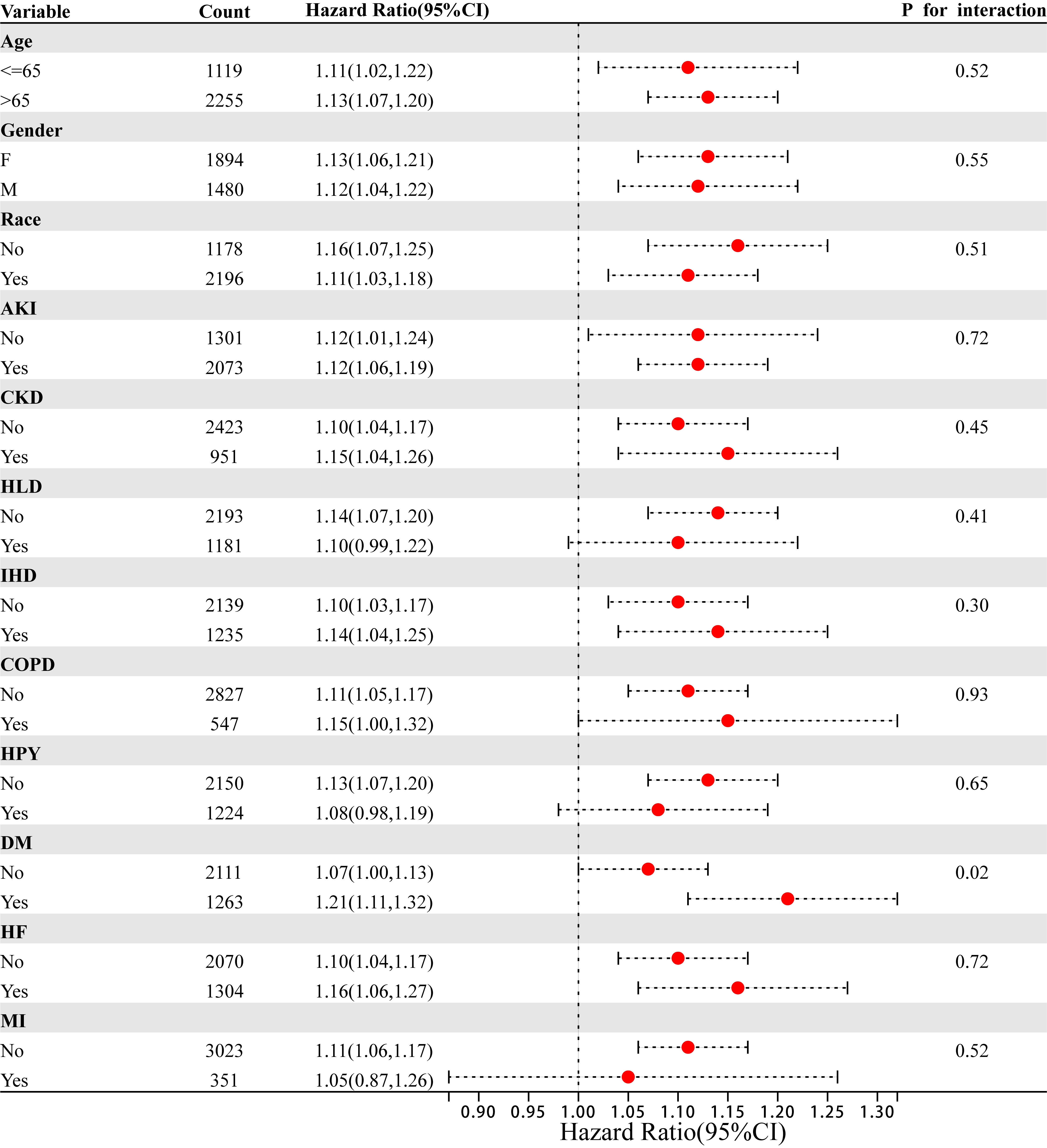


Fig S1: Subgroup and interaction analysis of the relationship between RAR and short-term mortality in patients with urinary sepsis in ICU
