# Supplementary material for: Red cell distribution width to albumin ratio predicts short-term mortality in urosepsis: a dual-cohort study
Source: Front Nutr. 2026 Feb 10;13:1709663. doi: 10.3389/fnut.2026.1709663 (PMC12929096; doi:10.3389/fnut.2026.1709663)
Supplement: Supplementary file 3 [file Table_1.docx]

Table S1: Baseline data of patients in ICU Dead (Discovery queue)

|  | **ALL** | **Survivor** | **No-survivor** | **P value** |
| --- | --- | --- | --- | --- |
|  | ***N=3374*** | ***N=2861*** | ***N=513*** |  |
| Age | 70.4 (15.1) | 70.0 (15.2) | 72.9 (14.2) | <0.001 |
| Gender: | 1480 (43.9%) | 1262 (44.1%) | 218 (42.5%) | 0.528 |
| Race: | 2196 (65.1%) | 1881 (65.7%) | 315 (61.4%) | 0.064 |
| Weight | 82.1 (25.6) | 82.1 (25.5) | 81.9 (26.0) | 0.871 |
| HPY: | 1224 (36.3%) | 1069 (37.4%) | 155 (30.2%) | 0.002 |
| AKI: | 2073 (61.4%) | 1681 (58.8%) | 392 (76.4%) | <0.001 |
| CKD: | 951 (28.2%) | 788 (27.5%) | 163 (31.8%) | 0.056 |
| DM: | 1263 (37.4%) | 1084 (37.9%) | 179 (34.9%) | 0.214 |
| HLD: | 1181 (35.0%) | 1041 (36.4%) | 140 (27.3%) | <0.001 |
| HF: | 1304 (38.6%) | 1091 (38.1%) | 213 (41.5%) | 0.161 |
| MI: | 351 (10.4%) | 289 (10.1%) | 62 (12.1%) | 0.202 |
| IHD: | 1235 (36.6%) | 1048 (36.6%) | 187 (36.5%) | 0.978 |
| COPD: | 547 (16.2%) | 453 (15.8%) | 94 (18.3%) | 0.179 |
| SOFA | 6.51 (3.59) | 6.22 (3.39) | 8.16 (4.16) | <0.001 |
| APSII | 57.0 (21.2) | 55.0 (20.2) | 67.9 (23.3) | <0.001 |
| SAPSII | 43.9 (13.5) | 42.7 (12.9) | 50.9 (14.2) | <0.001 |
| OASIS | 35.3 (8.37) | 34.9 (8.18) | 37.9 (8.94) | <0.001 |
| Charlson | 6.27 (2.90) | 6.11 (2.87) | 7.14 (2.93) | <0.001 |
| APACHEII | 21.2 (7.11) | 20.7 (6.98) | 23.9 (7.23) | <0.001 |
| HR | 91.1 (21.2) | 91.0 (21.2) | 91.8 (21.0) | 0.407 |
| NBPS | 121 (25.7) | 121 (25.6) | 116 (26.0) | <0.001 |
| NBPD | 67.9 (19.7) | 68.3 (19.4) | 65.7 (21.0) | 0.010 |
| RR | 20.1 (6.32) | 20.1 (6.32) | 20.3 (6.28) | 0.333 |
| Spo2 | 96.6 (4.53) | 96.6 (4.49) | 96.2 (4.72) | 0.028 |
| Hb | 10.2 (2.21) | 10.2 (2.21) | 9.93 (2.22) | 0.004 |
| PLT | 204 (118) | 206 (118) | 191 (120) | 0.011 |
| RDW | 16.0 (2.57) | 15.8 (2.44) | 16.9 (3.01) | <0.001 |
| RBC | 3.42 (0.78) | 3.44 (0.77) | 3.31 (0.82) | 0.001 |
| WBC | 13.6 (14.2) | 13.3 (13.4) | 15.4 (17.8) | 0.014 |
| ALB | 2.93 (0.61) | 2.96 (0.60) | 2.78 (0.67) | <0.001 |
| AG | 15.6 (4.73) | 15.4 (4.67) | 16.2 (5.00) | 0.001 |
| Ca | 8.29 (0.96) | 8.30 (0.96) | 8.25 (0.99) | 0.343 |
| Cl | 104 (7.94) | 104 (7.77) | 103 (8.83) | 0.066 |
| Glu | 154 (84.3) | 154 (84.7) | 150 (82.1) | 0.222 |
| K | 4.22 (0.80) | 4.20 (0.79) | 4.29 (0.81) | 0.026 |
| Na | 138 (6.72) | 139 (6.57) | 138 (7.49) | 0.223 |
| TCO2 | 24.1 (6.39) | 24.2 (6.37) | 23.2 (6.44) | 0.002 |
| Lac | 2.36 (1.96) | 2.29 (1.92) | 2.78 (2.17) | <0.001 |
| PCO2 | 41.9 (12.3) | 41.9 (12.3) | 41.7 (12.4) | 0.773 |
| PH | 7.35 (0.10) | 7.36 (0.10) | 7.34 (0.11) | 0.001 |
| PO2 | 119 (97.2) | 121 (98.7) | 107 (87.5) | 0.002 |
| INR | 1.67 (1.04) | 1.62 (0.99) | 1.91 (1.27) | <0.001 |
| PT | 18.1 (10.6) | 17.6 (10.0) | 20.7 (13.3) | <0.001 |
| PTT | 39.9 (24.4) | 39.1 (23.9) | 44.1 (26.8) | <0.001 |
| ALT | 130 (609) | 132 (632) | 120 (459) | 0.596 |
| AST | 218 (1030) | 219 (1051) | 214 (910) | 0.914 |
| TB | 2.07 (5.01) | 1.77 (4.23) | 3.73 (7.86) | <0.001 |
| CRE | 1.83 (1.71) | 1.81 (1.72) | 1.96 (1.65) | 0.055 |
| URE | 37.0 (29.0) | 35.6 (27.9) | 44.7 (33.5) | <0.001 |
| SA: | 2310 (68.5%) | 1910 (66.8%) | 400 (78.0%) | <0.001 |
| VP: | 2142 (63.5%) | 1736 (60.7%) | 406 (79.1%) | <0.001 |
| GC: | 1090 (32.3%) | 889 (31.1%) | 201 (39.2%) | <0.001 |
| Ventilation: | 2893 (85.7%) | 2448 (85.6%) | 445 (86.7%) | 0.525 |
| CRRT: | 326 (9.66%) | 234 (8.18%) | 92 (17.9%) | <0.001 |
| RAR | 5.75 (1.72) | 5.62 (1.64) | 6.45 (1.98) | <0.001 |
| RAR group: |  |  |  | <0.001 |
| Low | 1687 (50.0%) | 1500 (52.4%) | 187 (36.6%) |  |
| High | 1687 (50.0%) | 1361 (47.6%) | 326 (63.4%) |  |
