# Supplementary material for: Red cell distribution width to albumin ratio predicts short-term mortality in urosepsis: a dual-cohort study
Source: Front Nutr. 2026 Feb 10;13:1709663. doi: 10.3389/fnut.2026.1709663 (PMC12929096; doi:10.3389/fnut.2026.1709663)
Supplement: Supplementary file 4 [file Table_2.docx]

Table S2: Baseline data of patients in Hosp Dead (Discovery queue)

|  | **ALL** | **Survivor** | **No-survivor** | **P value** |
| --- | --- | --- | --- | --- |
|  | ***N=3374*** | ***N=2910*** | ***N=464*** |  |
| Age | 70.4 (15.1) | 69.9 (15.2) | 73.5 (14.1) | <0.001 |
| Gender: | 1480 (43.9%) | 1280 (44.0%) | 200 (43.1%) | 0.760 |
| Race: | 2196 (65.1%) | 1911 (65.7%) | 285 (61.4%) | 0.084 |
| Weight | 82.1 (25.6) | 82.2 (25.5) | 81.5 (26.2) | 0.615 |
| HPY: | 1224 (36.3%) | 1082 (37.2%) | 142 (30.6%) | 0.007 |
| AKI: | 2073 (61.4%) | 1718 (59.0%) | 355 (76.5%) | <0.001 |
| CKD: | 951 (28.2%) | 801 (27.5%) | 150 (32.3%) | 0.038 |
| DM: | 1263 (37.4%) | 1105 (38.0%) | 158 (34.1%) | 0.117 |
| HLD: | 1181 (35.0%) | 1051 (36.1%) | 130 (28.0%) | 0.001 |
| HF: | 1304 (38.6%) | 1107 (38.0%) | 197 (42.5%) | 0.078 |
| MI: | 351 (10.4%) | 291 (10.0%) | 60 (12.9%) | 0.066 |
| IHD: | 1235 (36.6%) | 1063 (36.5%) | 172 (37.1%) | 0.863 |
| COPD: | 547 (16.2%) | 462 (15.9%) | 85 (18.3%) | 0.208 |
| SOFA | 6.51 (3.59) | 6.24 (3.41) | 8.23 (4.15) | <0.001 |
| APSII | 57.0 (21.2) | 55.2 (20.3) | 68.0 (23.5) | <0.001 |
| SAPSII | 43.9 (13.5) | 42.8 (13.0) | 51.2 (14.1) | <0.001 |
| OASIS | 35.3 (8.37) | 34.9 (8.19) | 38.2 (8.90) | <0.001 |
| Charlson | 6.27 (2.90) | 6.13 (2.87) | 7.17 (2.94) | <0.001 |
| APACHEII | 21.2 (7.11) | 20.8 (6.99) | 24.0 (7.20) | <0.001 |
| HR | 91.1 (21.2) | 91.0 (21.2) | 91.5 (20.7) | 0.642 |
| NBPS | 121 (25.7) | 121 (25.6) | 116 (26.0) | <0.001 |
| NBPD | 67.9 (19.7) | 68.3 (19.5) | 65.4 (20.6) | 0.005 |
| RR | 20.1 (6.32) | 20.0 (6.31) | 20.4 (6.37) | 0.246 |
| Spo2 | 96.6 (4.53) | 96.6 (4.50) | 96.2 (4.72) | 0.050 |
| Hb | 10.2 (2.21) | 10.2 (2.20) | 10.0 (2.24) | 0.084 |
| PLT | 204 (118) | 205 (117) | 195 (121) | 0.075 |
| RDW | 16.0 (2.57) | 15.9 (2.51) | 16.8 (2.79) | <0.001 |
| RBC | 3.42 (0.78) | 3.44 (0.77) | 3.35 (0.83) | 0.031 |
| WBC | 13.6 (14.2) | 13.4 (13.8) | 15.2 (16.0) | 0.019 |
| ALB | 2.93 (0.61) | 2.96 (0.60) | 2.76 (0.64) | <0.001 |
| AG | 15.6 (4.73) | 15.5 (4.69) | 16.3 (4.87) | 0.001 |
| Ca | 8.29 (0.96) | 8.31 (0.97) | 8.20 (0.94) | 0.026 |
| Cl | 104 (7.94) | 104 (7.78) | 103 (8.87) | 0.098 |
| Glu | 154 (84.3) | 154 (84.8) | 149 (81.7) | 0.200 |
| K | 4.22 (0.80) | 4.20 (0.79) | 4.30 (0.82) | 0.016 |
| Na | 138 (6.72) | 139 (6.55) | 138 (7.69) | 0.252 |
| TCO2 | 24.1 (6.39) | 24.2 (6.37) | 23.1 (6.38) | <0.001 |
| Lac | 2.36 (1.96) | 2.29 (1.91) | 2.80 (2.21) | <0.001 |
| PCO2 | 41.9 (12.3) | 41.9 (12.3) | 41.7 (12.5) | 0.770 |
| PH | 7.35 (0.10) | 7.36 (0.10) | 7.34 (0.11) | <0.001 |
| PO2 | 119 (97.2) | 121 (98.6) | 108 (86.7) | 0.004 |
| INR | 1.67 (1.04) | 1.63 (0.99) | 1.91 (1.30) | <0.001 |
| PT | 18.1 (10.6) | 17.7 (10.0) | 20.6 (13.6) | <0.001 |
| PTT | 39.9 (24.4) | 39.2 (24.1) | 43.9 (26.2) | <0.001 |
| ALT | 130 (609) | 131 (627) | 129 (482) | 0.943 |
| AST | 218 (1030) | 217 (1042) | 230 (955) | 0.785 |
| TB | 2.07 (5.01) | 1.84 (4.46) | 3.53 (7.43) | <0.001 |
| CRE | 1.83 (1.71) | 1.81 (1.72) | 1.98 (1.66) | 0.037 |
| URE | 37.0 (29.0) | 35.7 (27.9) | 45.1 (33.7) | <0.001 |
| SA: | 2310 (68.5%) | 1950 (67.0%) | 360 (77.6%) | <0.001 |
| VP: | 2142 (63.5%) | 1777 (61.1%) | 365 (78.7%) | <0.001 |
| GC: | 1090 (32.3%) | 912 (31.3%) | 178 (38.4%) | 0.003 |
| Ventilation: | 2893 (85.7%) | 2487 (85.5%) | 406 (87.5%) | 0.274 |
| CRRT: | 326 (9.66%) | 246 (8.45%) | 80 (17.2%) | <0.001 |
| RAR | 5.75 (1.72) | 5.63 (1.66) | 6.44 (1.97) | <0.001 |
| RAR group: |  |  |  | <0.001 |
| Low | 1687 (50.0%) | 1516 (52.1%) | 171 (36.9%) |  |
| High | 1687 (50.0%) | 1393 (47.9%) | 294 (63.1%) |  |
