# Supplementary material for: Red cell distribution width to albumin ratio predicts short-term mortality in urosepsis: a dual-cohort study
Source: Front Nutr. 2026 Feb 10;13:1709663. doi: 10.3389/fnut.2026.1709663 (PMC12929096; doi:10.3389/fnut.2026.1709663)
Supplement: Supplementary file 5 [file Table_3.docx]

Table S3: Baseline data of patients in ICU Dead (Verify queue)

|  | **ALL** | **Survivor** | **No-survivor** | **P value** |
| --- | --- | --- | --- | --- |
|  | ***N=245*** | ***N=210*** | ***N=35*** |  |
| RAR | 5.63 (1.53) | 5.45 (1.32) | 6.74 (2.15) | 0.001 |
| Age | 72.2 (15.6) | 71.7 (15.7) | 75.4 (15.2) | 0.191 |
| Gender | 0.45 (0.50) | 0.45 (0.50) | 0.46 (0.51) | 0.918 |
| BMI | 27.3 (4.83) | 27.3 (4.83) | 27.2 (4.92) | 0.865 |
| HPY: | 99 (40.4%) | 90 (42.9%) | 9 (25.7%) | 0.084 |
| AKI: | 138 (56.3%) | 115 (54.8%) | 23 (65.7%) | 0.305 |
| CKD: | 64 (26.1%) | 52 (24.8%) | 12 (34.3%) | 0.327 |
| DM: | 94 (38.4%) | 82 (39.0%) | 12 (34.3%) | 0.727 |
| HF: | 97 (39.6%) | 78 (37.1%) | 19 (54.3%) | 0.083 |
| COPD | 0.14 (0.35) | 0.12 (0.32) | 0.26 (0.44) | 0.085 |
| SOFA | 6.18 (3.15) | 5.98 (2.97) | 7.37 (3.92) | 0.051 |
| APSII | 55.5 (18.6) | 53.5 (16.5) | 67.5 (25.4) | 0.003 |
| SAPII | 44.1 (12.6) | 42.8 (11.9) | 51.9 (14.3) | 0.001 |
| OASIS | 35.4 (7.93) | 35.1 (7.63) | 37.0 (9.50) | 0.258 |
| Charlson | 6.30 (2.75) | 6.06 (2.70) | 7.71 (2.67) | 0.001 |
| APACHEII | 20.9 (6.68) | 20.4 (6.37) | 23.7 (7.85) | 0.022 |
| HR | 90.3 (21.9) | 89.7 (21.7) | 94.1 (22.8) | 0.291 |
| NBPS | 122 (26.8) | 124 (26.1) | 113 (28.9) | 0.035 |
| NBPD | 69.5 (20.2) | 69.9 (19.5) | 66.7 (24.2) | 0.453 |
| RR | 20.3 (6.38) | 20.3 (6.31) | 20.2 (6.87) | 0.948 |
| Spo2 | 96.6 (3.91) | 96.7 (3.97) | 96.3 (3.55) | 0.510 |
| Hb | 10.3 (2.00) | 10.4 (1.99) | 9.77 (1.98) | 0.112 |
| PLT | 205 (121) | 207 (122) | 192 (117) | 0.494 |
| RDW | 15.9 (2.46) | 15.7 (2.40) | 16.9 (2.59) | 0.013 |
| RBC | 3.48 (0.75) | 3.51 (0.75) | 3.28 (0.76) | 0.097 |
| WBC | 14.1 (15.8) | 14.4 (16.9) | 12.7 (6.92) | 0.310 |
| ALB | 2.94 (0.59) | 2.98 (0.55) | 2.70 (0.75) | 0.037 |
| AG | 15.6 (4.43) | 15.5 (4.39) | 15.9 (4.68) | 0.639 |
| Ca | 8.39 (0.88) | 8.41 (0.85) | 8.28 (1.06) | 0.484 |
| Cl | 104 (7.48) | 104 (7.21) | 105 (8.98) | 0.488 |
| Glu | 154 (77.0) | 156 (75.6) | 142 (84.9) | 0.389 |
| K | 4.23 (0.74) | 4.22 (0.75) | 4.28 (0.73) | 0.641 |
| Na | 139 (6.47) | 139 (6.38) | 139 (7.12) | 0.906 |
| TCO2 | 23.4 (6.04) | 23.7 (6.04) | 22.0 (5.93) | 0.128 |
| Lac | 2.48 (1.96) | 2.43 (1.97) | 2.76 (1.85) | 0.344 |
| PCO2 | 40.0 (11.2) | 40.1 (11.4) | 39.4 (9.91) | 0.692 |
| PH | 7.36 (0.10) | 7.37 (0.10) | 7.34 (0.09) | 0.159 |
| PO2 | 120 (97.1) | 126 (99.6) | 87.4 (73.6) | 0.009 |
| INR | 1.63 (1.16) | 1.54 (0.82) | 2.18 (2.26) | 0.106 |
| PT | 17.7 (12.0) | 16.8 (8.25) | 23.5 (24.1) | 0.110 |
| PTT | 40.2 (25.9) | 39.2 (25.4) | 46.5 (28.6) | 0.158 |
| ALT | 153 (908) | 167 (979) | 71.1 (122) | 0.177 |
| AST | 201 (1010) | 219 (1088) | 98.4 (169) | 0.136 |
| TB | 1.67 (3.11) | 1.63 (3.01) | 1.88 (3.72) | 0.705 |
| CRE | 1.95 (1.97) | 1.89 (1.97) | 2.28 (1.96) | 0.284 |
| URE | 36.8 (28.7) | 34.7 (26.7) | 49.5 (36.2) | 0.025 |
| SA: | 155 (63.3%) | 133 (63.3%) | 22 (62.9%) | 1.000 |
| VP: | 147 (60.0%) | 123 (58.6%) | 24 (68.6%) | 0.351 |
