# Supplementary material for: Red cell distribution width to albumin ratio predicts short-term mortality in urosepsis: a dual-cohort study
Source: Front Nutr. 2026 Feb 10;13:1709663. doi: 10.3389/fnut.2026.1709663 (PMC12929096; doi:10.3389/fnut.2026.1709663)
Supplement: Supplementary file 6 [file Table_4.docx]

Table S4: Abbreviation table

| Abbreviation | English full name | Suggested unit/description |
| --- | --- | --- |
| AG | Anion Gap | mEq/L |
| AKI | Acute Kidney Injury | Diagnostic category |
| ALB | Albumin | g/dL |
| ALT | Alanine Aminotransferase | IU/L |
| APACHE II | Acute Physiology and Chronic Health Evaluation II | Score |
| APSII | Acute Physiology Score II | Score |
| AST | Aspartate Aminotransferase | IU/L |
| Ca | Calcium | mg/dL |
| Charlson | Charlson Comorbidity Index | Score |
| CKD | Chronic Kidney Disease | Diagnostic category |
| Cl | Chloride | mEq/L |
| COPD | Chronic Obstructive Pulmonary Disease | Diagnostic category |
| CRE | Creatinine | mg/dL |
| CRRT | Continuous Renal Replacement Therapy | Treatment modality |
| DM | Diabetes Mellitus | Diagnostic category |
| GC | Glucocorticoids | Binary (0/1) |
| Glu | Glucose | mg/dL |
| Hb | Hemoglobin | g/dL |
| HF | Heart Failure | Diagnostic category |
| HLD | Hyperlipidemia | Diagnostic category |
| HPY | Hypertension | Diagnostic category |
| HR | Heart Rate | beats/min |
| ICU dead | 28-Day ICU Mortality | Binary (0/1) |
| ICU 28 time | 28-Day ICU Length of Stay | days |
| IHD | Ischemic Heart Disease | Diagnostic category |
| INR | International Normalized Ratio | Ratio |
| K | Potassium | mEq/L |
| Lac | Lactate | mmol/L |
| MI | Myocardial Infarction | Diagnostic category |
| NBPD | Non-Invasive Blood Pressure (Diastolic) | mmHg |
| NBPS | Non-Invasive Blood Pressure (Systolic) | mmHg |
| OASIS | Oxford Acute Severity of Illness Score | Score |
| PCO₂ | Partial Pressure of Carbon Dioxide | mmHg |
| PH | pH Value | pH units |
| PLT | Platelet Count | K/μL |
| PO₂ | Partial Pressure of Oxygen | mmHg |
| PT | Prothrombin Time | seconds |
| PTT | Partial Thromboplastin Time | seconds |
| RAR | RDW to ALT Ratio | Ratio |
| RBC | Red Blood Cell Count | m/uL |
| RDW | Red Cell Distribution Width | % |
| RR | Respiratory Rate | insp/min |
| SA | Sedative Administration | Binary (0/1) |
| SAPII | Simplified Acute Physiology Score II | Score |
| SOFA | Sequential Organ Failure Assessment | Score |
| Spo₂ | Oxygen Saturation | % |
| TB | Total Bilirubin | mg/dL |
| TCO₂ | Total Carbon Dioxide | mEq/L |
| URE | Urea Nitrogen | mg/dL |
| VP | Vasopressor | Binary (0/1) |
| WBC | White Blood Cell Count | K/μL |
